# Cost-sensitive Bayesian control policy in human active sensing

**DOI:** 10.3389/fnhum.2014.00955

**Published:** 2014-12-03

**Authors:** Sheeraz Ahmad, He Huang, Angela J. Yu

**Affiliations:** ^1^Computer Science and Engineering, University of CaliforniaSan Diego, La Jolla, CA, USA; ^2^Cognitive Science, University of CaliforniaSan Diego, La Jolla, CA, USA

**Keywords:** active sensing, visual search, Bayesian model, markov decision processes, overt attention, saccadic eye movements

## Abstract

An important but poorly understood aspect of sensory processing is the role of *active sensing*, the use of self-motion such as eye or head movements to focus sensing resources on the most rewarding or informative aspects of the sensory environment. Here, we present behavioral data from a visual search experiment, as well as a Bayesian model of within-trial dynamics of sensory processing and eye movements. Within this Bayes-optimal inference and control framework, which we call C-DAC (Context-Dependent Active Controller), various types of *behavioral costs*, such as temporal delay, response error, and sensor repositioning cost, are explicitly minimized. This contrasts with previously proposed algorithms that optimize abstract statistical objectives such as anticipated information gain (Infomax) (Butko and Movellan, 2010) and expected posterior maximum (greedy MAP) (Najemnik and Geisler, 2005). We find that C-DAC captures human visual search dynamics better than previous models, in particular a certain form of “confirmation bias” apparent in the way human subjects utilize prior knowledge about the spatial distribution of the search target to improve search speed and accuracy. We also examine several computationally efficient approximations to C-DAC that may present biologically more plausible accounts of the neural computations underlying active sensing, as well as practical tools for solving active sensing problems in engineering applications. To summarize, this paper makes the following key contributions: human visual search behavioral data, a context-sensitive Bayesian active sensing model, a comparative study between different models of human active sensing, and a family of efficient approximations to the optimal model.

## 1. Introduction

The brain excels at performing sensory inference under conditions of uncertainty. One important tool the brain has at its disposal is *active sensing*, the ability to utilize self-motion in order to allocate sensing resources toward the most rewarding or informative aspects of the environment. Most prominent models of sensory processing presume *passiveness* in data collection (Simoncelli and Heeger, 1998; Riesenhuber and Poggio, 1999; Lewicki, 2002; Wilson and Mainen, 2006), considering only how to represent or compute with given inputs, and not how to actively intervene in the input collection process itself, especially with respect to behavioral goals or environmental constraints. Having a formal understanding of active sensing is not only important for advancing neuroscientific progress but also for engineering applications, such as developing context-sensitive, interactive artificial sensing systems.

The most well-studied aspect of human active sensing is saccadic eye movements (Yarbus, 1967). While most existing models have focused on bottom-up saliency factors related to image statistics (Koch and Ullman, 1987; Itti and Koch, 2000; Lee and Yu, 2000; Rao et al., 2002; Itti and Baldi, 2006), empirical data have long indicated eye movements to be under the influence of a variety of cognitive factors (Yarbus, 1967; Land and Lee, 1994; Rayner, 1998; Land and Hayhoe, 2001; Henderson, 2007), such as prior knowledge about target location (He and Kowler, 1989), temporal onset (Oswal et al., 2007), and reward probabilities (Roesch and Olson, 2003). Models that assume saccadic eye movements to be passive or automatic responses to the visual scene sidestep the computational consequences of saccadic decisions, and thus cannot explain how behavioral goals and environmental context ought to influence eye movements. In part to overcome this limitation, the notion of saliency has been reframed probabilistically and dynamically in terms of maximizing the future informational gain (Infomax) given current beliefs about the visual scene (Lee and Yu, 2000; Itti and Baldi, 2006; Butko and Movellan, 2010). Separately, it has also been proposed that saccades may be dynamically chosen to maximize the greedy probability of finding the target in one time step (Greedy MAP) (Najemnik and Geisler, 2005).

While both Infomax and Greedy MAP brought a new level of sophistication to the modeling of eye movements—in particular, representing sensory processing as iterative Bayesian inference, quantifying the information gain of different saccade choices, and incorporating knowledge about sensory noise—they are still limited in some key respects: (1) they optimize abstract computational quantities that do not directly relate to behavioral goals (e.g., speed and accuracy) or task constraints (e.g., the cost of moving sensors from one location to another); (2) there is no explicit representation of time in these algorithms, and thus no principled means of trading off fixation duration or number of fixations with performance.

In this work, we propose a behaviorally-grounded inference and control framework for active sensing, which we call C-DAC (Context-Dependent Active Controller). In contrast to Infomax and Greedy MAP, which optimize abstract statistical objectives, C-DAC explicitly optimizes behaviorally defined objectives. Specifically, we assume that the observer aims to optimize a context-sensitive objective function that takes into account behavioral costs such as temporal delay, response error, and the cost of switching from one sensing location to another. C-DAC uses this compound objective to plan eye movements adaptively, based on a continually updated, statistically optimal (Bayesian) representation of the history of observations. This framework allows us to derive behaviorally optimal procedures for making decisions about (1) where to acquire sensory inputs, (2) when to move from one observation location to another, and (3) how to negotiate the exploration-exploitation tradeoff between collecting additional data vs. terminating the observation process. In what follows, we cast our problem as a Partially Observable Markov Decision Process (POMDP) since the state (location of the targets) are hidden and have to be inferred on the basis of accumulated evidence from sampling (with eye movements). Since the influence of action choices on the hidden state variable (e.g., visual object location) is trivial, we can convert the POMDP into a *belief Markov Decision Process*, or a Markov Decision Process whose states are the (observable) belief states and the action choices non-trivially affect future belief states.

Complementing the modeling work, we also present data from a visual search experiment, in which subjects are required to find a designated target stimulus amongst distractor stimuli, in the presence of sensory noise. A critical experimental manipulation is that some locations are more likely to contain the target than others, during each block of trials. As reported elsewhere (Yu and Huang, 2014), we found that subjects' first fixation choice on each trial is consistent with having internalized spatial statistics of target location based on experienced target location in previous trials. In this work, we analyze how this prior information exerts control over the within-trial, fine-temporal dynamics of eye movements and perceptual processing. We find that humans exhibit a certain confirmation bias on this task—a tendency to favor the most likely location in processing speed and perceptual choice. We compare the various models with human behavior, in terms of fixation choice and duration, to investigate whether human behavior is better explained by taking into account task-specific considerations, as in C-DAC, or whether it is sufficient to optimize an abstract, and thus generic, statistical goal, like that of Infomax or Greedy-MAP.

Due to its contextual sensitivity, C-DAC may also improve on current algorithms in engineering applications, such as the digital eye (Butko and Movellan, 2010), where Infomax has been applied. However, the standard numerical solution for C-DAC is computationally intense—it scales exponentially with the number of potential target locations, making it impractical for most real-world applications. We there for propose two kinds of approximations schemes for C-DAC to sidestep its computational complexity: (1) approximate dynamic programming based on low-dimensional parametric and non-parametric approximation of the value function, (2) one-step look-ahead myopic approximation of value function that sidesteps dynamic programming. These approximations retain context-sensitivity while significantly reducing computational complexity. We will examine in this paper how these approximations compare to C-DAC, as well as areas for further investigations.

## 2. Methods

In the following, we model active sensing as solving a sequential (Markov) decision process problem, where the observer can control both the location where the data is collected, and the amount of data collected in each location. Although we use language specific to visual search for concreteness, the framework discussed below easily generalizes to a broad range of active sensing problems. As a Markov decision process problem, active sensing consists of two separable computational components, an *inference* component that extracts a useful representation of the external environment based on the observed stream of noisy data, and a *decision* component that decides how to maintain or modify or terminate the sensing process as a function of this extracted representation. In the following, we begin with a discussion of the inference process, common to C-DAC and Infomax, and then discuss the decision policy for each of C-DAC and Infomax. We then discuss various possible approximations to the C-DAC policy, and conclude with a description of the visual search experiment. We exclude Greedy-MAP from our analysis since it resembles a random policy for the simple visual search problem we consider (see Supplementary Material for details).

### 2.1. Inference

We assume the observer starts with a prior belief over the true target location *s* (out of *k* possible locations), based on prior searching encounters (trials in the experimental setting), and then updates his/her belief about target location via Bayes' rule, upon receiving each new observation *x_t_*. The observation noise is assumed to be i.i.d. conditioned on target location *s* and sensing (fixation) location λ_*t*_. Thus, if we denote the sequence of fixation locations up to time *t* as λ_*t*_: = {λ_1_, …, λ_*t*_}, the sequence of observations up to time *t* as **x**_*t*_: = {*x*_1_, …, *x_t_*}, and the belief state at time *t* as **p**_*t*_: = (*P*(*s* = 1|**x**_*t*_; λ_*t*_), …, *P*(*s* = *k*|**x**_*t*_; λ_*t*_)), then the belief-update rule according to Bayes' theorem is:

(1) $$\begin{array}{l} p_{t}^{i}=P\left(s=i|x_{t};\lambda_{t}=j,\lambda_{t\;-\;1}\right)\propto p\left(x_{t}|s=i;\lambda_{t}=j\right)\\ \;P\left(s=i|x_{t\;-\;1};\lambda_{t\;-\;1}\right)=f_{i,j}(x_{t})p_{t\;-\;1}^{i} \end{array}$$

where *f_i,j_*(*x_t_*) is the likelihood function, and **p**_0_ the prior belief distribution over target location.

### 2.2. C-DAC decision policy

For the decision policy, *C-DAC* optimizes the mapping from the belief state to the action space (continue, switching to one of the other sensing locations, stopping and report the target location) with respect to a behavioral cost function. If the target is at location *s*, and the observer declares it to be at location δ, after spending τ units of time and making *n*_τ_ number of switches between potential target locations, then the total cost incurred is given by:

(2) $$l(\tau ,\delta ;s)=c\tau +c_{s}n_{\tau }+1_{\left\{\delta \;\neq \;s\right\}}$$

where *c* is the cost per unit time, *c_s_* is the cost per switch, and cost of making a wrong response is 1 (since we can always make one of the costs to be unity via normalization). For any given policy π (mapping belief state to action), the expected cost is $L_{\pi }:=c𝔼[\tau ]+c_{s}𝔼[n_{s}]+P(\delta \neq s)$. At any time *t*, the observer can either choose to stop and declare one of the locations to be the target, or choose to continue and look at location λ_*t* + 1_. Thus, the expected cost associated with stopping and declaring location *i* to be the target is:

(3) $$\begin{array}{l} {\bar{Q}}_{t}^{i}(p_{t};\lambda_{t}):=𝔼\left[l(t,i;s)|p_{t};\lambda_{t}\right]=ct+c_{s}n_{t}\\ \;+\;𝔼\left[1_{\left\{i\;\neq \;s\right\}}\right]=ct+c_{s}n_{t}+\left(1-p_{t}^{i}\right) \end{array}$$

And the minimum expected cost for continuing sensing at location *j* is:

(4) $$\begin{array}{l} Q_{t}^{\;j}(p_{t}=p;\lambda_{t}):=c(t+1)+c_{s}\left(n_{t}+1_{\left\{j\;\neq \;\lambda_{t}\right\}}\right)\\ \;+\underset{{}_{\tau^{\prime },\delta ,\lambda_{\tau^{\prime }}}}{\min}𝔼\left[l(\tau^{\prime },\delta ;s)|p_{0}=p;\lambda_{1}=j\right] \end{array}$$

The value function *V*(**p**, *i*), or the expected cost incurred following the optimal policy (π^*^), starting with the prior belief **p**_0_ = **p** and initial observation location λ_1_ = *i*, is:

(5) $$V(p,i):=\underset{{}_{\tau ,\delta ,\lambda_{\tau }}}{\min}𝔼\left[l(\tau ,\delta ;s)|p_{0}=p;\lambda_{1}=i\right]$$

Then the value function satisfies the following recursive relation (Bellman, 1952), and the action that minimizes the right hand side is the optimal action π^*^(**p**, *k*):

(6) $$\begin{array}{l} V(p,k)=\min\left(\left(\underset{i}{\min}{\bar{Q}}_{1}^{i}(p;k)\right),\left(\underset{j}{\min}Q_{1}^{i}(p;k)\right)\right)\\ \;=\min\left(\underset{i}{\min}\left(1-p^{i}\right),\underset{j}{\min}\left(c+c_{s}1_{\left\{j\neq k\right\}}+𝔼\left[V(p^{\prime },j)\right]\right)\right) \end{array}$$

where *k* is the fixation location and **p**′ is the random variable denoting the achievable belief states in the next time-step. The equation can be interpreted as a *forward simulation* till the stopping horizon, where for any belief state **p**, at *t* = 1, the observer can either stop and declare a location to be the target, or sample one more observation, followed by the optimal action (which in turn can be stop or continue). Notice that the horizon is finite because the cost of continuation keeps increasing with time and eventually (depending on the parameters) it would exceed the cost of stopping, making the stop action the optimal choice. Furthermore, the optimal decision policy is a stationary policy—the value function depends only on the belief state and observation location at the time the decision is to be taken, and not on time *t per se*. The exact solution can be obtained using dynamic programming for the continuous belief state. In practice, we discretize the belief state space, initialize the value function over this space (any choice would do, we use the stopping cost, 0 is another common choice), and iterate Equation 6 until convergence.

### 2.3. Infomax decision policy

The objective of Infomax is to maximize long-term future information gain (or minimize cumulative future entropy of the posterior belief state) (Butko and Movellan, 2010). Thus, the action-values, value function, and the resultant policy can be formulated as follows in the Markov decision process framework:

$$\begin{array}{l} Q_{I}(p_{t},j)=\sum_{t^{\prime }=t+1}^{T}𝔼[H(p_{t^{\prime }})|p_{t};\lambda_{t+1}=j];\\ \;V_{I}(p_{t})=\underset{j}{\min}Q_{I}(p_{t},j);\\ \;\lambda_{t+1}=\;\underset{j}{\arg\min}Q_{I}(p_{t},j) \end{array}$$

Infomax policy (and value function) does not depend on current location, since the action costs *Q_I_* (**p**_*t*_, *j*) only concern with the entropy of the belief state in the next time step, a random variable that depends on the current belief state and next location but not on the current fixation location. Furthermore, Infomax does not directly prescribe when to stop, since there are only continuation actions and no *stopping action*. A general heuristic used for such strategies is to stop when the confidence in one of the locations being the target (the belief about that location) exceeds a certain threshold, which is a free parameter challenging to set for any specific problem. We augment infomax policy using the bound developed in Theorem 1, which yields a stopping region that is a provable subset of the optimal stopping region for C-DAC, and which is sensitive to observation noise parameterized by β and the cost of time parameterized by *c*.

### 2.4. Approximate decision policy

We present two approximate decision policies that limit the complexity of dynamic programming (Equation 6) by either approximating the value function using a low-dimensional basis function representation, or by forgoing value iteration altogether by using a myopic horizon to assess action values.

### 2.5. Value function approximation

**Figure 1:**
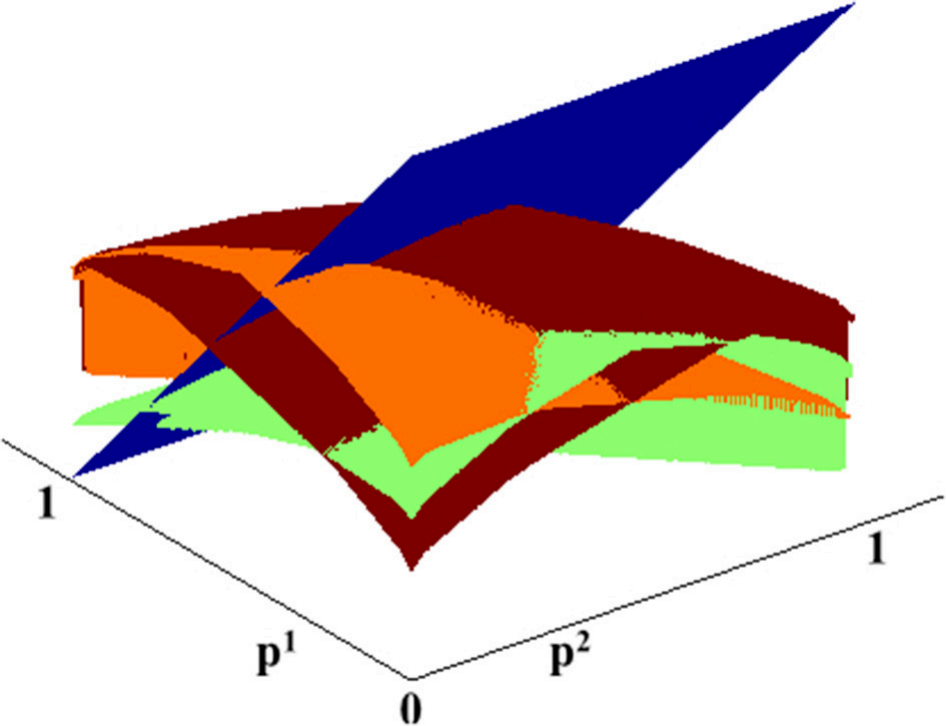
**C-DAC Q-factors for different actions**. Blue: stop and declare. Green: fixate location 1. Orange: fixate location 2. Brown: fixate location 3.

As shown in Figure 1, the Q-factors (Equation 3, 4) as well as the resulting value function (Equation 6) are *smooth and concave*, making them amenable to low dimensional approximations. At each step, we find a low dimensional representation of the value function, and use that for the update step of the value iteration algorithm. Specifically, instead of re-computing the value function at each grid point, here we generate a large number of samples uniformly on the belief state space, compute a new estimate of the value function at those locations, and then extrapolate the value function to everywhere by improving its parametric fit.

The first low-dimensional approximation we consider is Radial Basis Function (RBF) approximation (Buhmann, 2003), which has been proposed to characterize neural encoding and computation in the visual pathways (Poggio, 1990), and also having been applied to many engineering applications. The overall scheme for approximating value functions with RBF proceeds using the following sequence of algorithmic steps:

1. Generate M RBFs, centered at {μ_*i*_}^*M*^_*i* = 1_, with fixed $\sigma :\phi (p)=\frac{1}{\sigma {\left(2\pi \right)}^{k/2}}e^{\frac{||p\;-\;\mu_{i}|{|}^{2}}{2\sigma^{2}}}$
2. Generate *m* random points from belief space, **p**.
3. Initialize {*V*(**p**_*i*_)}^*m*^_*i* = 1_ with the stopping costs.
4. Find minimum-norm **w** from: *V*(**p**) = Φ(**p**) **w**.
5. Generate new *m* random belief state points (**p**′).
6. Evaluate required *V* values using current **w**.
7. Update *V*(**p**′) using value iteration.
8. Find a new **w** from *V*(**p**′) = Φ(**p**′) **w**.
9. Repeat steps 5 through 8, until *w* converges.

While we adopt a Gaussian kernel function, other functional forms are also possible, e.g., multiquadratic $\left(\phi (p)=\sqrt{1+\epsilon ||p-\mu_{i}|{|}^{2}}\right)$, inverse-quadratic(ϕ(**p**) = (1 + ϵ||**p** − μ_*i*_||^2^)^−1^), and thin plate spline (ϕ(**p**) = ||**p** − μ_*i*_||^2^ ln ||**p** − μ_*i*_||). We have implemented some of these for our problem without observing significant deviations from the RBF results (data not shown).

The RBF approximation requires setting several parameters (number, location and width of bases), which can be impractical for large problems, when there is little or no information available about the properties of the true value function. We thus also implement a non-parametric variation of the algorithm, whereby we use Gaussian Process Regression (GPR) (Williams and Rasmussen, 1996) to estimate the value function (step 4, 6, and 8). In addition, we also implement GPR with hyperparameter learning (Automatic Relevance Determination, ARD), thus obviating the need to pre-set model parameters.

The approximations lead to considerable computational savings. The complexity of the RBF approximation is *O* (*k*(*mM* + *M*^3^)), for *k* sensing locations, *m* random points chosen at each step, and *M* bases. For the GPR approximation, the complexity is *O*(*kN*^3^), where *N* is the number of points used for regression. In practice, all the approximation algorithms we consider converge rapidly (under 10 iterations), though we do not have a proof that this holds for a general case.

### 2.6. Myopic approximation

This approximation attempts to optimize the contextual cost of C-DAC, but only for one step in the future. In other words, the planning is based on the inherent assumption that the next action is the last non-terminal action permissible (or considered), and so the goal is to minimize the cost incurred in this single step. The actions available are, thus, stop and declare the current location as the target, or choose another sensing location before stopping. Similar to Equation 6, we can write the value function as:

(7) $$V(p,k)=\min\left(\left(1-p^{k}\right),\underset{j}{\min}\left(c+c_{s}1_{\left\{j\neq k\right\}}+\underset{{}_{l_{j}}}{\min}\left(1-𝔼[p^{l_{j}}]\right)\right)\right)$$

where *j* indexes the possible sensing locations, and *l_j_* indexes the possible stopping actions for the sensing location *j*.

Note that the value function computation does not involve any recursion, just a comparison between simple-to-compute action values for different actions. Furthermore, this online policy only needs to be calculated for the current belief state, making the computational complexity to be constant in time.

If we define the stopping region as the subset of the belief space where it is optimal to stop the search and declare the target, it can be seen that this myopic policy overestimates the size of the stopping region as compared to the C-DAC policy: if there is only one step left, it is never optimal to continue looking at the same location, since such an action would not lead to any improvement in expected accuracy, but incur a unit cost of time *c*. We present a novel theoretical result that gives an inner bound of the stopping region, which is sensitive to the sampling cost *c* and the signal-to-noise ratio of the sensory input.

### 2.7. Stopping threshold bound

**Theorem 1**. *Assuming that the observations are binary and Bernoulli distributed*:

$$\begin{array}{l} f_{i,j}(x)=p(x|s=i;\lambda =j)=1_{\left\{i=j\right\}}\beta^{x}{\left(1-\beta \right)}^{1-x}\\ \;+\;1_{\left\{i\neq j\right\}}{\left(1-\beta \right)}^{x}\beta^{1-x} \end{array}$$

If p^*^ is the solution of the equation:

$$p\frac{\left(2\beta -1)(1-p\right)}{\beta p+(1-\beta )(1-p)}=c$$

where c is the cost per unit time as defined in section C-DAC Decision Policy, then for all **p**^i^ > p^*^, the optimal action is to stop and declare location *i* under the cost formulation of C-DAC.

*Proof*. The cost incurred for collecting each new sample is *c*. Therefore, stopping is optimal when the improvement in belief from collecting another sample is less than the cost incurred to collect that sample. Formally, stopping and choosing *i* is optimal for the corresponding belief **p**^*i*^ = *p* when: 
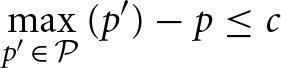
 where 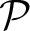 is the set of achievable beliefs starting from *p*. Furthermore, if we solve the above equation for equality, to find p^*^, then by problem construction, it is always optimal to stop for *p* > p^*^ (stopping cost (1 − *p*) < (1 − p^*^)). Given the likelihood function *f*_*s*, λ_(*x*) (Equation 8), we can use Equation 1 to simplify the above relation to:

$$p\frac{\left(2\beta -1)(1-p\right)}{\beta p+(1-\beta )(1-p)}=c$$

                     □

### 2.8. Experimental design

In this subsection, we detail our visual search experiment. We collected the data from eleven subjects, recruited from the UCSD undergraduate students (five females). Subjects first performed a random-dot coherent motion direction discrimination task (Britten et al., 1992) training session and achieved an accuracy exceeding 75% for 12%-coherence stimuli, before continuing onto the main experiment. In the main visual search experiment, subjects must identify one of the three random-dot stimulus patches as the target (left-moving for five subjects, right-moving for six subjects), the other two being distractor stimuli moving in the opposite direction. Subjects began each trial by fixating a central cross, then sequentially fixated one or more stimulus patches until pressing a space bar, which indicated that the last viewed stimulus was the chosen target. The three stimulus patches were circular and equidistant from the central cross, rotationally symmetrically positioned at non-cardinal angles. In the 1:1:1 condition (2 blocks), the target appeared in the three locations with equal likelihood on each trial; in the 1:3:9 locations (6 blocks, one of each possible configuration), the target appeared in the three locations with correspondingly biased probabilities. The order of the eight blocks (six biased blocks and two uniform ones, 90 trials per block) were randomized for each subject. Before the main experiment, subjects experienced 3 practice blocks: respectively, they consisted of 30, 40, and 40 trials, each with target location distribution drawn randomly from the configuration in the main experiment (2/8 probability of a 1:1:1 block, 1/8 probability of each of the 6 1:3:9 blocks). The random-dot motion coherence of the three blocks were 30%, 20%, and 12%, respectively. A target identification accuracy of 80% had to be reached in the first two practice blocks, or else the same block has to be repeated; similarly, in the third practice block, an accuracy of 68% had to be reached before the subject could proceed to the main experiment. Other than experiencing practice blocks with similar statistics as in the main experiment, subjects did not receive explicit instructions on the spatial distribution of target location.

**Figure 2:**
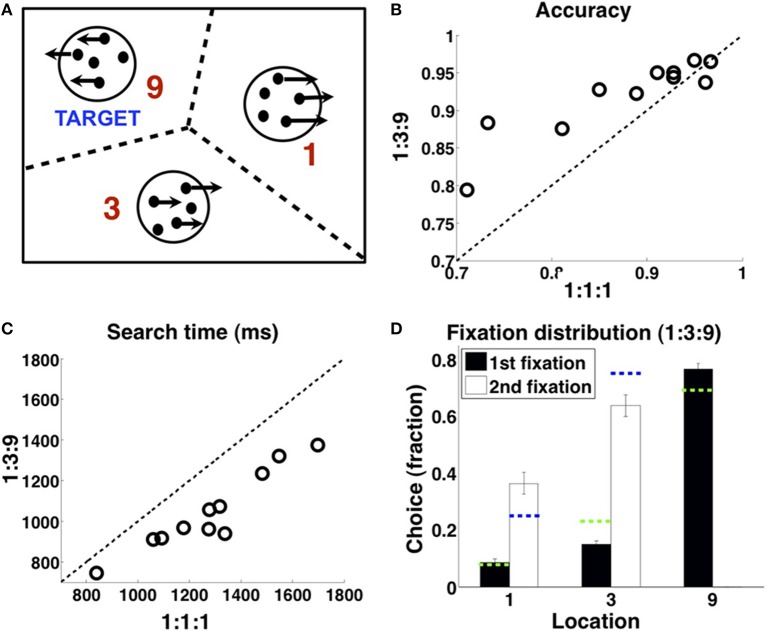
**Experimental design and data**. **(A)** On each trial, two of the random-dot stimuli are distractors, one is the target; subjects must find the target (see Methods). **(B)** Subjects are more accurate in finding the target in the 1:3:9 condition than the 1:1:1 condition, and **(C)** faster. **(D)** 1:3:9 condition, allocation of fixation location on first fixation (black), and second fixation when subjects first fixated the 9 location and found that it was not the target (white), averaged over all subjects. Green dashed lines indicate the matching probabilities on the first fixation, (1/13, 3/13, 9/13); blue dashed lines indicate matching probabilities on the second fixation, (1/4, 3/4). *n*=11. Errorbars: s.e.m. across subjects. Adapted with permission from Yu and Huang (2014). Copyright © 2014 by the American Psychological Association.

The gaze-contingent display only revealed a motion stimulus in the fixated location, with the remainder replaced by a central dot; boundaries for fixation determining which stimulus was shown at any given time are as shown in Figure 2A. Subjects' eye movements were monitored using a SR Research Eyelink 1000 eye tracker. A timing bar on the left side of the screen indicated time elapsed since onset of first stimulus fixation, first decreasing in length (green) until 8 s elapsed, and then growing in length in the opposite direction (red) at the same rate, though subjects were told that points were deducted indefinitely in proportion to their total response time (12.5/s). At the end of each trial, subjects were shown the true target location and the total points gained/lost for that trial: 100–12.5 × (seconds taken to respond) −25× (number of fixation switches, 0 if only one patch fixated) ± 50 (+ if final response correct, —if incorrect). Subjects were paid at the end of the experiment proportionally to the total points earned, which were calibrated so as to award the average subject about USD 10 an hour.

All subjects had normal or corrected vision. They were excluded from the analyses if they achieved less than 50% accuracy in the main experiment (lower than in the practice blocks), or showed unusually large first fixation spatial bias (> 2 standard deviations away in Kullback-Leibler divergence from the population mean distribution of first fixation, in the 1:3:9 condition). This lead to the exclusion of one out of twelve participants.

## 3. Results

We first briefly describe the visual search task (see Methods, also Yu and Huang, 2014), before delving into the experimental findings and comparison to the various models. In the task (Figure 2A), subjects must find a target stimulus (random-dot motion stimulus moving in a certain direction) in one of three possible locations, with the other two locations containing distractors (random-dot motion stimulus moving in the opposite direction). In the 1:3:9 condition, the target location is biased among the three options with 1:3:9 odds. In the 1:1:1 control condition, the target appears in the three locations with equal probability on each trial. To eliminate the complications associated with the spatiotemporal dynamics of covert attention, which we cannot measure directly, the display is gaze-contingent: only the fixated stimulus is visible at any given time, with the other two stimuli being replaced by two small dots located at the center of the stimulus patches. Subjects receive feedback about true target location on each trial after making their choice, as well as their choice accuracy, search duration, and number of switches; they are encouraged through a point-based reward function to be fast, accurate, and efficient with the number of fixation switches (see Methods).

As shown in Figure 2 (adapted from Yu and Huang, 2014), human subjects appeared to internalize and exploit the spatial statistics to locate the target stimulus more accurately (Figure 2B) and more rapidly (Figure 2C). Subjects were more accurate in finding the target in the 1:3:9 condition than the 1:1:1 condition (one-sided *t*-test, *p* < 0.01), and faster at finding the target than in the 1:3:9 condition than the 1:1:1 condition (*p* < 0.001). Underlying this performance improvement was a prioritized search strategy that favors the more probable locations as a fixation choice (Figure 2D). For first fixation, subjects preferentially fixated the 9 location over the 3 location (*p* < 0.0001), which in turn was favored over the 1 location (*p* < 0.01). For second fixation, on trials in which the first fixation was at 9 and that was *not* the target, subjects then favored the 3 location over the 1 location (*p* = 0.005).

**Figure 3:**
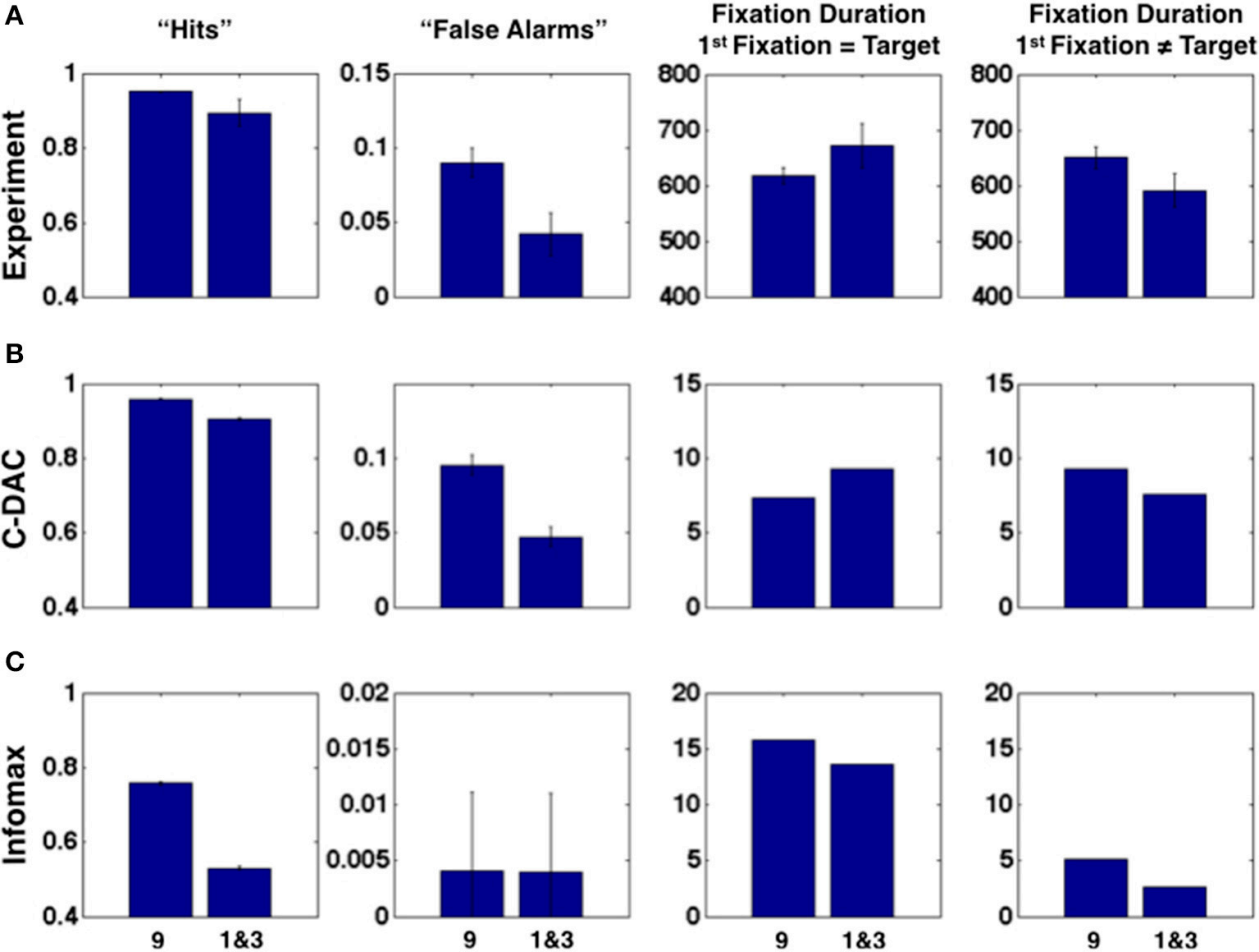
**The influence of prior spatial knowledge on sensory processing and decision-making, in humans and models**. **(A)** In terms of perceptual choice, subjects are more likely to choose the most probable target location (“9”) than the less probable locations (“1 and 3”) as containing the target, whether it actually contains the target (“hits,” first column) or not (“false alarms,” second column). In terms of fixation duration (milliseconds), subjects are faster to accept the most probable location, compared to the less probable locations, as containing the target, when the fixated location contains the target (third column); they are slower to accept the most probable location, as compared to the less probable locations, as *not* containing the target, when the fixated location does *not* contain the target (fourth column). All data included in the plots are from trials where the subject first fixated the labeled location. The data for the “1” and “3” locations are combined due to the relatively low incidence of subjects' first fixating these locations. **(B)** C-DAC reproduces the same pattern of behavior as human subjects. Fixation duration in number of simulation steps. **(C)** Infomax fails to reproduce the same pattern of behavior as human subjects. Simulation settings: (*c, c_s_*, β) = (0.005, 0.1, 0.68).

Subjects' first-fixation choices reflect a dynamically updated Bayesian prior belief, related to the true underlying spatial distribution of target location as well as incidental stimulus history experienced in the recent past (Yu and Huang, 2014). In this work, we focus on how that prior belief on each trial modulates sensory processing and perceptual decision-making within each trial, as reflected in the fine temporal dynamics of fixation patterns and durations. As shown in Figure 3A, subjects exhibit a certain confirmation bias in their fixation patterns. When viewing the same sensory input, subjects were more likely to identify the stimulus as a target if it is in the 9 location rather than in the 1 or 3 locations, thus exhibiting both a higher hit and false alarm rate in the 9 location compared to the other locations. Moreover (column 3 and 4), on correct trials, subjects appear to need *less* sensory evidence to confirm a stimulus as the target but *more* evidence to reject it as a distractor in the more probable location: subjects spent less time viewing a *target* stimulus in the 9 patch than the 1 and 3 patches; conversely, they spent longer viewing a *distractor* stimulus in the 9 patch than the 1 and 3 patches. To isolate the effect of prior spatial information on perceptual processing, and thus any indirect influence of prior information on motor response (evident in Figure 2D), which in turn may affect sensory processing, all the data shown here (Figure 3A) only include trials where subjects first fixated the labeled patch, i.e., the 9 patch in the “9” column and the 1 or 3 patch in the “1 and 3” column. Data for 1 and 3 patches were combined together due to the small number of trials in which subjects first fixated one of these locations.

We consider two different models for human eye movements: C-DAC and Infomax. We do not present results for Greedy MAP, which is highly sub-optimal and behaves like a random policy for this task (see Supplementary Material). In the simulations, observation noise is assumed to be binary (Bernoulli distributed). Separate simulations using Gaussian observation noise leads to very similar value functions and policies for different settings of the problem (results not shown); thus, for simplicity, we use the simpler binary noise model to illustrate the main points in the paper. C-DAC uses iterative Bayesian inference to update the belief about the target location based on sequential sensory data, and makes a decision about how long to view a location before switching to another one or choosing a terminal response, so that the overall *behavioral cost* is minimized (a linear combination of the cost of time, the cost of making a switch among locations, and the cost of making an incorrect response). The Infomax formulation also employs Bayesian belief updates about target location, but makes fixation decisions so as to maximize the anticipated cumulative future entropy reduction about the target location. The parameters for C-DAC were selected by first transposing the experimental parameters into appropriate units and then performing a grid search in a narrow region around these initial values to find the best fitting candidates.

**Figure 4:**
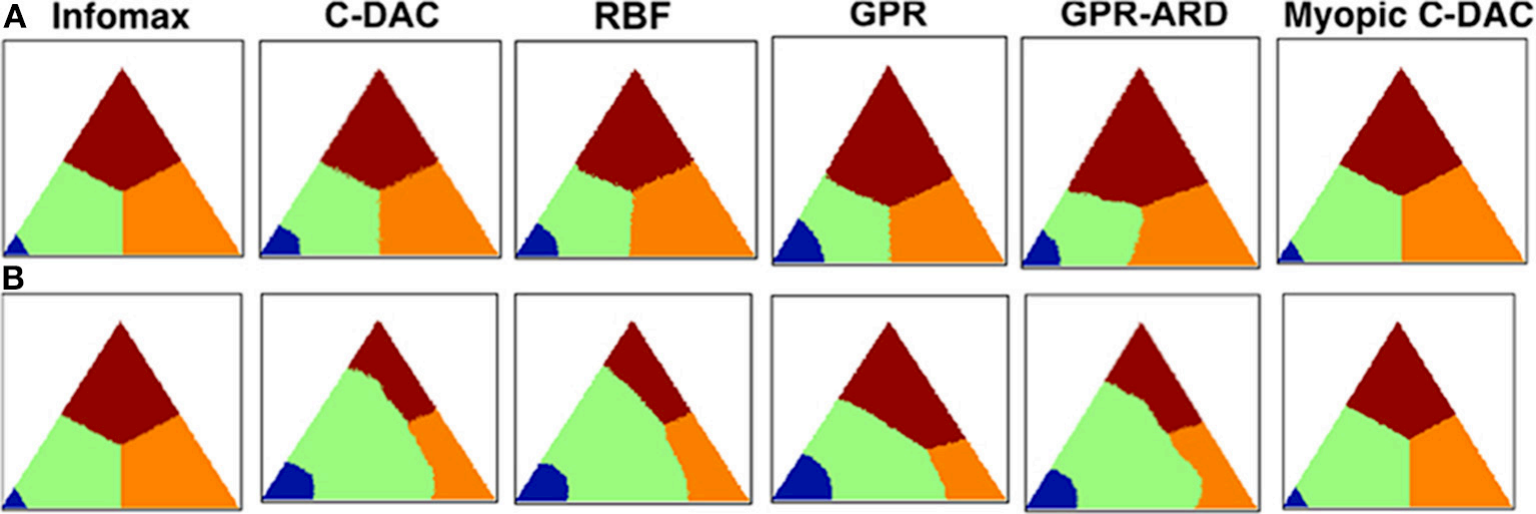
**Decision policies as a function of switching cost**. **(A)** No switch-cost environment: (*c, c_s_*, β) = (0.1, 0, 0.9). **(B)** Switch-cost environment: (*c, c_s_*, β) = (0.1, 0.1, 0.9). Triangle represents the belief state space, (*p*^1^, *p*^2^), under affine transform for visual symmetry. Color-coding denotes the action prescribed by each policy for different values of the belief state vector. Blue: stop and declare location 1 as target. Green: fixate location 1. Orange: fixate location 2. Brown: fixate location 3. All policies are deterministic (see Methods).

We find that the confirmation bias phenomena exhibited by humans are captured by C-DAC (Figure 3B), but not by Infomax (Figure 3C). This is due to Infomax's insensitivity to the cost of switching sensing location, parameterized by *c_s_* in C-DAC. As seen in Figure 4, the inability to incorporate switching cost makes the Infomax decision policy quite different from the optimal policy (C-DAC). In C-DAC, larger *c_s_* induces increasingly more asymmetric decision regions, whereby the optimal policy becomes more reluctant to switch away from the currently fixated location, despite sensory evidence pointing to the contrary. This would come into play when the observer is fixated in a stimulus location that does *not* contain the target (Figure 3, column 4), as the switching cost accentuates a reluctance to switch away from this location, especially when it is *a priori* perceived to be more likely to contain the target. Separately, larger *c_s_* also increases the size of the stopping region (Figure 4), as the incentive of switching to other location just to “make sure” the currently fixated location contains the target diminishes, and the optimal policy instead chooses to terminate observations and select the current location as the target even for intermediate levels of confidence. This is why C-DAC has shorter fixation duration on target trials for the “9” location, as the higher prior probability, combined with the preliminary sensory evidence, is sufficient to push the optimal policy into the stopping region, whereas the lower prior confidence accorded to “1” and “3” locations does not succeed in doing so. In contrast, Infomax in its original formulation (Butko and Movellan, 2008) has no principled stopping policy at all, and in its augmented form here (Theorem 1) is still insensitive to *c_s_* and moreover under-estimates the size of the stopping region (see Figure 4). Thus, not only does Infomax fixate longer than C-DAC, in general, before stopping on the target trials (Figure 3, column 3), but the higher prior for the “9” location is insufficient, combined with sensory data, to push it earlier into the stopping region than for the “1” and “3” locations. Altogether, the results suggest that humans, like C-DAC, modify the fine temporal dynamics of their visual search behavior in a way that is apparently sensitive to the switch cost, which may arise from intrinsic temporal or energetic cost associated with saccades, and/or due to the experimental design, which levies 25 points per fixation switch in the task (see Methods).

### 3.1. Model predictions

**Figure 5:**
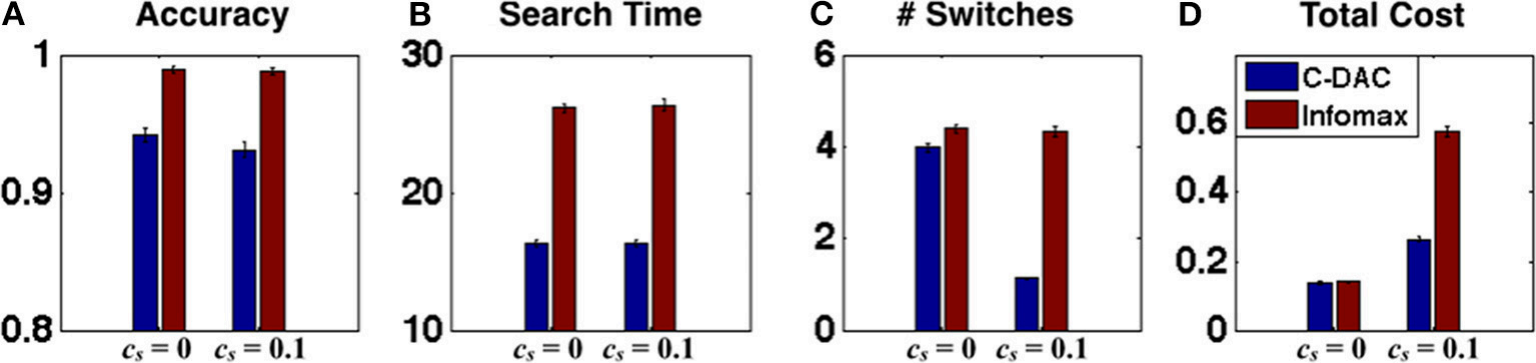
**Behavioral predictions as a function of switching cost**. **(A)** Accuracy, **(B)** search time, **(C)** number of switches, and **(D)** total behavioral cost, for C-DAC and Infomax for two environments (*c, c_s_*, β) = (0.005, 0, 0.68) and (0.005, 0.1, 0.68). For *c_s_* > 0, C-DAC reduces the number of switches and incurs overall less behavioral cost.

In this section, we describe future experiments that can further differentiate the various eye movement models, by considering scenarios in which Infomax and C-DAC are predicted to have particularly divergent behavior. We showed in the previous section that the switch cost may play a key role in modulating human visual search behavior. We therefore use model simulations to predict how behavior should change depending on whether the switching between fixation locations is penalized or not (e.g., in our experiment design, this can be done by changing the number of points charged for fixation switch, which results in greater monetary penalty). As can be seen in Figure 4, explicitly manipulating switch cost leads to rather different control policies. This should presumably lead to rather different behavioral outcomes as well. As shown in Figure 5A, for the case *c_s_* = 0, the accuracy of C-DAC is poorer as compared to Infomax, because the threshold used for Infomax (based on Theorem 1) is more conservative (thus stopping and declaration happens at higher confidence, leading to higher accuracy), but C-DAC takes less time (Figure 5B) and fewer switches (Figure 5C) to reach the decision. Looking at the various behavioral costs, we see that although C-DAC loses in accuracy, it makes up through other measures, leading to slightly lower total cost (Figure 5D). For the case when *c_s_* = 0.1, the accuracy and search time for both policies are relatively comparable to the case with *c_s_* = 0. However, C-DAC has a notable advantage in terms of number of switches, while the number of switches remains unchanged for Infomax (Figures 5A,B). This case exemplifies the context-sensitivity of C-DAC, as it reduces number of switches when switching becomes more costly (Figure 5C). When all these costs are combined, we see that C-DAC incurs a lower cost than Infomax (Figure 5D). These observations can be used to aid the design of future experiments, to gain a fuller understanding of human eye movement behavior.

**Figure 6:**
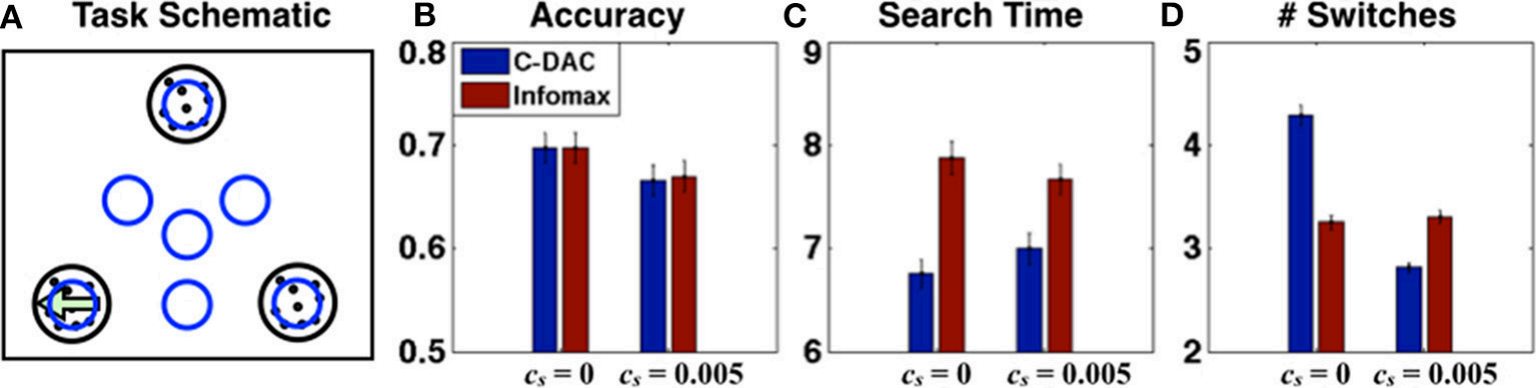
**Visual search with peripheral vision**. **(A)** Task schematics: agent fixates one of the blue circle regions at any given time, the target (left-moving dots) is in one of the black circle regions. **(B)** Accuracy, **(C)** Search time and **(D)** number of switches for C-DAC and Infomax for two environments (*c*, β1, β_2_, β_3_, β_4_) = (0.05, 0.62, 0.6, 0.55, 0.5), *c_s_* = 0 and *c_s_* = 0.005. C-DAC adjusts the time steps and number of switches depending on the environment, taking a little longer but reducing number of switches when effort has cost.

Another way to extend our work is to include peripheral vision in future experiments. Here, we model a simple task with peripheral vision (see Figure 6A), whereby the observer can saccade to intermediate locations that give information about more than one stimulus (either on the edge between two stimuli, or in the center of the triangle delimited by all three stimuli), but gives noisier information than when a stimulus is directly fixated. This is particularly relevant given experimental observations, that humans not only fixate the most probable target locations but sometimes also center-of-gravity locations that are intermediate among two or more target locations (Findley, 1982; Zelinsky et al., 1997).

Formally, we need the notion of gradually degraded peripheral vision, such that the quality of sensory information decreases at greater spatial distance away from the fovea. For example, consider the task of Figure 6A, whose fixation space consists of 7 elements, *L* = {*l*_1_, *l*_2_, *l*_3_, *l*_12_, *l*_23_, *l*_13_, *l*_123_}, where the first three actions correspond to fixating one of the three target locations, the next three to fixating midway between two target locations, and the last to fixating the center of all three. We parameterize the quality of peripheral vision by augmenting the observations to be three-dimensional, (*x*^1^, *x*^2^, *x*^3^), corresponding to the three simultaneously viewed locations. We assume that each *x_i_* is generated by a Bernoulli distribution favoring 1 if it is the target, and 0 if it is not, and its magnitude (absolute difference from 0.5) is greatest when observer directly fixates the stimulus, and smallest when the observer directly fixates one of the other stimuli.

We use 4 parameters to characterize the observations (1 > β_1_ > β_2_ > β_3_ > β_4_ > = 0.5). So, when the agent is fixating one of the potential target locations (*l*_1_, *l*_2_, or *l*_3_), it gets an observation from the fixated location (parameter β_1_ or 1 − β_1_ depending on whether it is the target or a distractor), and observations from the non-fixated locations (parameter β_4_ or 1 − β_4_ depending on whether they are a target or a distractor). Similarly, for the midway locations (*l*_12_, *l*_23_, or *l*_13_), the observations are received for the closest locations (parameter β_2_ or 1 − β_2_ depending on whether they are a target or a distractor), and from the farther off location (parameter β_4_ or 1 − β_4_ depending on whether it is the target or a distractor). Lastly, for the center location (*l*_123_), the observations are made for all three locations (parameter β_3_ or 1 − β_3_ depending on whether they are a target or a distractor). Furthermore, since the agent can now look at locations that cannot be target, we relax the assumption that the agent must look at a particular location before choosing it, allowing the agent to stop at any location and declare the target.

For performance comparison in terms of behavioral output, we again investigate two scenarios: (1) no switch cost, (2) with switch cost. The threshold for infomax is set so that the accuracies are matched to facilitate fair comparison. For all simulations, the algorithm starts with uniform prior (**p** = (1/3, 1/3, 1/3)) and initial fixation at the center (location *l*_123_), while the true target location is uniformly distributed. Figures 6B–D show the accuracy, number of time steps, and number of switches respectively for both scenarios. We do not show the total cost here (as in Figure 5D), because for this case, C-DAC matches or outperforms Infomax on all relevant criteria for both parameter settings, leading to a trivially lower cost. Note, however, that C-DAC makes more switches for *c_s_* = 0 (Figure 6D), which makes sense since switches incur no cost while search time does, and search time can potentially be reduced by allowing more switches. However, when switch cost is added (*c_s_* = 0.005), C-DAC significantly reduces number of switches (Figure 6D), whereas infomax lacks this ability to adapt to a changed environment.

### 3.2. Approximate policies

The active sensing framework proposed here is formally a variant of POMDP (Partially Observable Markov Decision Process), whereby the state space (target location) is hidden but the state dynamics is trivial as it does not depend on the action choice. Although there is a rich body of literature on approximate solutions of POMDP (e.g., Lagoudakis and Parr, 2003; Powell, 2007; Kaplow, 2010) for both general and application-specific scenarios, most are inappropriate for dealing with the problem here, as they are more aimed at solving problems with non-trivial state transitions and without low-dimensional sufficient statistics.

Equivalently, our problem can be treated as a fully observable Markov decision process problem, whereby the (observable) states are belief states and the transitions are known (via Bayesian belief updates). The dynamical programming equation (Equation 6) yields a numerical algorithm for computing the optimal decision policy, by defining an initial “guess” of the value function over the belief state space, and then using dynamic programming (Equation 6) to improve the value function until it converges to the true value function and the corresponding optimal decision policy. In practice, to use the dynamic programming equation (Equation 6), one needs to either assume a finite number of states, or use a parametric value function. Since our states represent belief states and are thus infinite (uncountably so, in fact), and the value function can be of arbitrary complexity, some approximation is needed. The standard approximation is to discretize the belief state space (Powell, 2007), which is known to be asymptotically optimal in the limit of arbitrarily fine gridding (Lovejoy, 1991) However, it has the twin disadvantages of introducing extra noise, as well as being exponentially expensive in representation and computation costs as a function of the number of dimensions in the state space, i.e., the number of possible target locations in the visual search task, or the number of possible hypotheses in a general active sensing problem. In particular, when there are *k* possible target locations, a uniform grid of size *n* has *O*(*kn*^*k* − 1^) cells.

In this paper we have proposed two classes of approximate control policies that limit the complexity of the value function: (1) low-dimensional parametric and non-parametric approximation of the Q-factors, and (2) myopic approximation of the value function. These approximations retain context-sensitivity while significantly reducing computational complexity. We discuss how these approximations compare to C-DAC, as well as their limitations that suggest further investigation.

Figure 4 shows different policies for a behavioral task on two environments: (*c, c_s_*, β) = (0.1, 0.1, 0.9) and (0.1, 0.1, 0.9). Each point on the plot corresponds to a belief state, i.e., the belief (expressed as probability) about which location has the target. For example, the belief state (*p*^1^, *p*^2^) translates to: probability that the target is at location 1 is *p*^1^, that it is at location 2 is *p*^2^, and that it is at location 3 is (1 − *p*^1^ − *p*^2^). There is a different policy plot for each location since the fixation strategy depends on current fixation location, but we only show the policy looking at location 1 since others are rotationally symmetric to this one. The color encodes the action prescribed by the policy at any given belief state, such that blue stands for stop and declare the current location as target, green for fixate location 1, orange for fixate location 2 and brown for fixate location 3. For example, as expected from any sensible policy, if the belief state is (1, 0) while looking at location 1, the suggested action is to stop and declare it as the target. Although the belief state is continuous, we discretize over a grid of size 201 for implementation. Note that for C-DAC (Figure 4B), the green region is larger compared to orange or brown signifying that the policy prescribes looking at location 1 even when the belief about target being at location 1 is somewhat low. This makes sense, since there is a cost associated with switching between locations, so the policy will choose switching only when the belief gets below a certain threshold.

We note that the parameter that accentuates the major difference between C-DAC and Infomax is the switch cost, *c_s_*, since its introduction leads to a shift in action boundaries. Unlike Infomax, the approximations we introduce can be seen to be sensitive to this cost and can modulate their switching boundaries. The boundary shift is less pronounced in Myopic C-DAC because of its greedy nature which only allows for incorporating one switch at the most, whereas in C-DAC and other approximations, a trial can potentially have multiple switches, leading to accumulating cost. In addition, Infomax does not adjust the size of its stopping boundary, unlike C-DAC and its parametric approximations. We used 49 Gaussian kernel bases for RBF (Radial Basis Functions) approximation, centered so as to uniformly grid the belief space (σ^2^ = 0.05), and 200 points to approximate the value function over. GPR (Gaussian Process Regression) approximation also uses 200 points to approximate the value function, and the parameters used are length scale 1, signal variance 1 and noise variance 0.1. GPR with ARD (Automatic Relevance Determination) uses 200 points as well and learns the parameters simultaneously with the value function.

## 4. Discussion

In this paper, we proposed a Bayes-risk minimization framework for active sensing, C-DAC, which optimizes behaviorally relevant objectives in expectation, such as speed, accuracy, and switching efficiency, and can reproduce the confirmation bias. We also presented a novel visual search experiment that involves finding a target stimulus amongst distractors, whereby the spatial distribution of the target stimulus is biased toward certain locations. We found that the fixation and choice behavior of subjects is modulated by top-down factors, specifically the likelihood of a particular location containing the target. Subjects were found to exhibit a certain confirmation bias—the tendency to systematically favor a location that is a priori judged more likely to contain the target, compared to another location less likely to contain the target, even in the face of identical sensory input and motor state. C-DAC was able to reproduce this confirmation bias. In contrast, Infomax, a policy that aims to optimize statistical objectives of task demands and ignores behavioral constraints (e.g., cost of time and switch), falls short. We augmented Infomax by introducing a stopping policy, which takes into account the time or sampling cost *c*, but that still did not sufficiently alleviate the context-insensitivity of Infomax. This is most likely due to both a sub-optimal incorporation of sampling cost and an intrinsic lack of sensitivity toward switching cost.

While C-DAC does a good job of reproducing human behavior, at least based on the experimental data presented here, we note that this does not necessarily imply that the brain implements C-DAC exactly. In particular, solving C-DAC exactly using dynamic programming requires a representational complexity that scales exponentially with the dimensionality of the search problem (i.e., the number of possible target locations), thus making it an impractical solution for more natural and complex problems faced daily by humans and animals. However, it is possible that the underlying computations can be approximated efficiently, making the formulation psychologically more plausible. We proposed different approximate algorithms that reduce the computational complexity of C-DAC in its original formulation. The low dimensional approximations provide a very good approximation to C-DAC, but these are still offline and global, requiring the computation of the policy for all possible belief states before engaging in the task. On the other hand, the myopic approximation to C-DAC we proposed scales linearly with search dimensionality, by eschewing a globally optimal solution that must be computed and maintained offline, in favor of an online, approximately and locally optimal solution. However, this myopic algorithm does not approximate C-DAC as well as the low-dimensional Q-factor approximations. One way to improve the myopic algorithm is to find better approximations to the switching and stopping boundaries without having to undergo the computationally expensive value iteration procedure, since these together completely characterize any decision policy. Our simulations (not shown here) suggest that there might be a systematic, monotonic relationship between the decision boundaries and the different cost parameters. We proposed one such bound on the stopping boundary here, and other approximate bounds have been proposed for similar problems (Naghshvar and Javidi, 2013). An alternative direction for exploring approximations to the optimal control problem is the recently proposed notion of “active inference," which casts the control problem as a pure inference problem and replaces value functions with a desired “belief state" regarding the consequences of actions, thus enabling the use of approximate inference techniques (e.g., variational) to solve the control problem (see Friston et al., 2012 for an introduction to active inference).

Another direction for future work is to do model comparison by comparing model predictions to individual human behavior on a trial-to-trial basis. The Experimental Prediction section describes some experimental settings and model predictions that could be particularly illuminating for differentiating the various models. It is worth noting that while the approximate policies are computationally simpler than the optimal model (and thus neurally more plausible), they are in fact more complex for fitting to human behavior, as they have additional model parameters (for parameterizing the approximation) on top of any parameters related to environmental statistics or cost functions, which all the policies share. This is an important point to keep in mind for experimental design and model fitting in future investigations.

In conclusion, although there is a rich body of literature on related problems in the application domain, our formulation stands out for its principled approach to incorporating contextual elements into the problem of active sensing. In general, the framework proposed here has the potential for not only applications in visual search, but a host of other problems, ranging from active scene categorization to active foraging. Its flexibility and robustness to different environments makes the framework an appealing choice for a variety of active sensing and related problems.

## Funding

This material is based upon work supported in part by the U. S. Army Research Laboratory and the U. S. Army Research Office under contract/grant number W911NF1110391.

### Conflict of interest statement

The authors declare that the research was conducted in the absence of any commercial or financial relationships that could be construed as a potential conflict of interest.

## References

[B1] Bellman R. (1952). On the theory of dynamic programming. Proc. Natl. Acad. Sci. 38, 716–719. 10.1073/pnas.38.8.716 16589166 PMC1063639

[B2] Britten K. H. Shadlen M. N. Newsome W. T. Movshon J. A. (1992). The analysis of visual motion: a comparison of neuronal and psychophysical performance. J. Neurosci. 12, 4745–4765. 1464765 10.1523/JNEUROSCI.12-12-04745.1992 PMC6575768

[B3] Buhmann M. (2003). Radial Basis Functions: Theory and Implementations, Vol. 12 New York, NY: Cambridge University Press.

[B4] Butko N. J. Movellan J. R. (2008). I-POMDP: an infomax model of eye movement, in Proceedings of the International Conference on Development and Learning (ICDL) (Monterey, CA).

[B5] Butko N. J. Movellan J. R. (2010). Infomax control of eyemovements. IEEE Trans. Auton. Ment. Dev. 2, 91–107 10.1109/TAMD.2010.2051029

[B6] Findley J. M. (1982). Global visual processing for saccadic eye movements. Vision Res. 22, 1033–1045. 10.1016/0042-6989(82)90040-2 7135840

[B7] Friston K. Samothrakis S. Montague R. (2012). Active inference and agency: optimal control without cost functions. Biol. Cybern. 106, 523–541. 10.1007/s00422-012-0512-8 22864468

[B8] He P. Y. Kowler E. (1989). The role of location probability in the programming of saccades: implications for “center-of-gravity” tendencies. Vision Res. 29, 1165–1181. 10.1016/0042-6989(89)90063-1 2617863

[B9] Henderson J. M. (2007). Regarding scenes. Curr. Dir. Psychol. Sci. 16, 219–222 10.1111/j.1467-8721.2007.00507.x

[B10] Itti L. Baldi P. (2006). Bayesian surprise attracts human attention, in Advances in Neural Information Processing Systems, Vol. 19 (Cambridge, MA: MIT Press), 1–8. 18834898

[B11] Itti L. Koch C. (2000). A saliency-based search mechanism for overt and covert shifts of visual attention. Vision Res. 40, 1489–1506. 10.1016/S0042-6989(99)00163-7 10788654

[B12] Kaplow R. (2010). Point-Based POMDP Solvers: Survey and Comparative Analysis. Ph.D. thesis, McGill University, Montreal, QC.

[B13] Koch C. Ullman S. (1987). Shifts in selective visual attention: towards the underlying neural circuitry, in Matters of Intelligence (Netherlands: Springer), 115–141. 3836989

[B14] Lagoudakis M. Parr R. (2003). Least-squares policy iteration. J. Mach. Learn. Res. 4, 1107–1149.

[B15] Land M. F. Hayhoe M. (2001). In what ways do eye movements contribute to everyday activities? Vision Res. 41, 3559–3565. 10.1016/S0042-6989(01)00102-X 11718795

[B16] Land M. F. Lee D. N. (1994). Where we look when we steer. Nature 369, 742–744. 10.1038/369742a0 8008066

[B17] Lee T. S. Yu S. (2000). An information-theoretic framework for understanding saccadic behaviors, in Advances in Neural Information Processing Systems, Vol. 12, eds Solla S. A. Leen T. K. Muller K.-R. (Cambridge, MA: MIT Press), 834–840.

[B18] Lewicki M. S. (2002). Efficient coding of natural sounds. Nat. Neurosci. 5, 356–363. 10.1038/nn831 11896400

[B19] Lovejoy W. (1991). Computationally feasible bounds for partially observed markov decision processes. Oper. Res. 39, 162–175 10.1287/opre.39.1.162

[B20] Naghshvar M. Javidi T. (2013). Active sequential hypothesis testing. Ann. Stat. 41, 2703–2738 10.1214/13-AOS1144

[B21] Najemnik J. Geisler W. S. (2005). Optimal eye movement strategies in visual search. Nature 434, 387–391. 10.1038/nature03390 15772663

[B22] Oswal A. Ogden M. Carpenter R. H. S. (2007). The time course of stimulus expectation in a saccadic decision task. J. Neurophysiol. 97, 2722–2730. 10.1152/jn.01238.2006 17267751

[B23] Poggio T. (1990). A theory of how the brain might work. Cold Spring Harb. Symp. Quant. Biol. 55, 899–910. 10.1101/SQB.1990.055.01.084 2132866

[B24] Powell W. (2007). Approximate Dynamic Programming: Solving the curses of dimensionality, Vol. 703 Wiley-Interscience.

[B25] Rao R. P. Zelinsky G. J. Hayhoe M. M. Ballard D. H. (2002). Eye movements in iconic visual search. Vision Res. 42, 1447–1463. 10.1016/S0042-6989(02)00040-8 12044751

[B26] Rayner K. (1998). Eye movements in reading and information processing: 20 years of research. Psychol. Bull. 124, 372–422. 10.1037/0033-2909.124.3.372 9849112

[B27] Riesenhuber M. Poggio T. (1999). Hierarchical models of object recognition in cortex. Nat. Neurosci. 2, 1019–1025. 10.1038/14819 10526343

[B28] Roesch M. R. Olson C. R. (2003). Impact of expected reward on neuronal activity in prefrontal cortex, frontal and supplementary eye fields and premotor cortex. J. Neurophysiol. 90, 1766–1789. 10.1152/jn.00019.2003 12801905

[B29] Simoncelli E. P. Heeger D. J. (1998). A model of neuronal responses in visual area mt. Vision Res. 38, 743–761. 10.1016/S0042-6989(97)00183-1 9604103

[B30] Williams C. K. I. Rasmussen C. E. (1996). Gaussian processes for regression, in Advances in Neural Information Processing Systems 8, eds Touretzky D. S. Mozer M. C. Hasselmo M. E. (Cambridge, MA: MIT Press), 514–520.

[B31] Wilson R. I. Mainen Z. F. (2006). Early events in olfactory processing. Annu. Rev. Neurosci. 29, 163–201. 10.1146/annurev.neuro.29.051605.112950 16776583

[B32] Yarbus A. F. (1967). Eye Movements and Vision. New York, NY: Plenum Press.

[B33] Yu A, J. Huang H. (2014). Maximizing masquerading as matching: statistical learning and decision-making in choice behavior. Decision 1, 275–287 10.1037/dec0000013

[B34] Zelinsky G. J. Rao R. P. Hayhoe M. M. Ballard D. H. (1997). Eye movements reveal the spatiotemporal dynamics of visual search. Psychol. Sci. 8, 448–453 10.1111/j.1467-9280.1997.tb00459.x

