# Supplementary material for: Cost-sensitive Bayesian control policy in human active sensing
Source: Front Hum Neurosci. 2014 Dec 3;8:955. doi: 10.3389/fnhum.2014.00955 (PMC4253738; doi:10.3389/fnhum.2014.00955)
Supplement: Supplementary file 1 [file DataSheet1.PDF]

Frontiers in Human Neuroscience

Original Research
29 November 2014

Supplementary Material: Cost-Sensitive Bayesian Control Policy in Human Active Sensing

Sheeraz Ahmad [1,*], He Huang [2] and Angela J. Yu,[2]

[1] *University of California San Diego, Computer Science and Engineering, La Jolla , CA, USA*
[2] *University of California San Diego, Cognitive Science, La Jolla , CA, USA*

Correspondence*:
Sheeraz Ahmad
University of California San Diego, Computer Science and Engineering, La Jolla , CA, USA, sahmad@ucsd.edu

GREEDY MAP POLICY: ANALYTICAL SOLUTION

The Greedy MAP policy suggests that agents should try to maximize the expected one-step look-ahead probability of finding the target. So, the reward associated with fixating location j at time $t+1$ is:

$$
\begin{aligned}
R^g(\mathbf{p}_t, j) &= \mathbb{E}_{x_{t+1}}[\max_i P(s=i|\mathbf{x}_t, x_{t+1}, \boldsymbol{\lambda}_t, \lambda_{t+1}=j)] \\
&= \sum_{x_{t+1}\in\{0,1\}} P(x_{t+1}|\lambda_{t+1}=j, \mathbf{x}_t, \boldsymbol{\lambda}_t,)[\max_i P(s=i|\mathbf{x}_t, x_{t+1}, \boldsymbol{\lambda}_t, \lambda_{t+1}=j)] \\
&= \sum_{x_{t+1}\in\{0,1\}} \left[\sum_k P(x_{t+1}|\lambda_{t+1}=j, \mathbf{x}_t, \boldsymbol{\lambda}_t, s=k) P(s=k|\lambda_{t+1}=j, \mathbf{x}_t, \boldsymbol{\lambda})\right] \left[\max_i (p^i_{t+1})\right] \\
&= \sum_{x_{t+1}\in\{0,1\}} \left[\sum_k P(x_{t+1}|\lambda_{t+1}=j, s=k) P(s=k|\mathbf{x}_t, \boldsymbol{\lambda})\right] \left[\max_i \frac{f_{i,j}(x_{t+1})p^i_t}{\sum_l f_{s,l}(x_{t+1})p^l_t}\right] \quad \text{(from eq. 1)} \\
&= \sum_{x_{t+1}\in\{0,1\}} \left[\sum_k f_{k,j}(x_{t+1})p^k_t\right] \left[\max_i \frac{f_{i,j}(x_{t+1})p^i_t}{\sum_l f_{l,j}(x_{t+1})p^l_t}\right] \\
&= \sum_{x_{t+1}\in\{0,1\}} \max_i f_{i,j}(x_{t+1})p^i_t \\
&= \sum_{x_{t+1}\in\{0,1\}} \max_i \left\{\left[\mathbf{1}_{\{i=j\}}\beta^{x_{t+1}}(1-\beta)^{1-x_{t+1}} + \mathbf{1}_{\{i\neq j\}}(1-\beta)^{x_{t+1}}\beta^{1-x_{t+1}}\right] p^i_t\right\} \quad \text{(Bernoulli pmf)}
\end{aligned}
$$

For the belief state, $\mathbf{p}_t$, the Greedy MAP policy is given by:

$$\lambda^g(\mathbf{p}_t) = \operatorname*{argmax}_j R^g(\mathbf{p}_t, j)$$

In order to demonstrate the policy plot, we consider the region where $p_t^1 > p_t^2 > p_t^3$, since these observations can then be generalized based on symmetry. Further, consider the three sub-regions:

Sub-region I. $(1-\beta)p_t^1 > \beta p_t^2$
For all $\mathbf{p}_t$ in this region, we get:

$$R^g(\mathbf{p}_t, 1) = R^g(\mathbf{p}_t, 2) = R^g(\mathbf{p}_t, 3) = p_t^1$$

Since all three rewards are same, there is no preference for the next fixation as per the Greedy MAP policy.

Sub-region II. $(1-\beta)p_t^1 < \beta p_t^2$ and $(1-\beta)p_t^1 > \beta p_t^3$
The rewards are:

$$R^g(\mathbf{p}_t, 1) = R^g(\mathbf{p}_t, 2) = \beta(p_t^1 + p_t^2)$$
$$R^g(\mathbf{p}_t, 3) = p_t^1$$

So, $R^g(\mathbf{p}_t, 1) = R^g(\mathbf{p}_t, 2) > R^g(\mathbf{p}_t, 3)$, and Greedy MAP policy is to fixate either location 1 or 2.

Sub-region III. $(1-\beta)p_t^1 < \beta p_t^2$ and $(1-\beta)p_t^1 < \beta p_t^3$
The rewards are:

$$R^g(\mathbf{p}_t, 1) = R^g(\mathbf{p}_t, 2) = \beta(p_t^1 + p_t^2)$$
$$R^g(\mathbf{p}_t, 3) = \beta(p_t^1 + p_t^3)$$

Again, $R^g(\mathbf{p}_t, 1) = R^g(\mathbf{p}_t, 2) > R^g(\mathbf{p}_t, 3)$, and Greedy MAP policy is to fixate either location 1 or 2.

Fig. 1(A) shows the policy plot for sub-regions I, II and III where blue denotes fixate 1, 2 or 3 and yellow denotes fixate 1 or 2. Also, based on the symmetry, the policy plot on the simplex is shown in Fig. 1(B) such that green corresponds to fixate 2 or 3 and orange fixate 3 or 1. This corresponds closely with simulations, as can be seen in Fig. 1(C), which shows one instance of the Greedy MAP policy – there are regions where it recommends that all locations are equally good, and similarly regions where two of the locations are equally good. The simulated policy also shows the stopping region (blue) which is an augmentation so that the algorithm has a confidence threshold about when to stop.

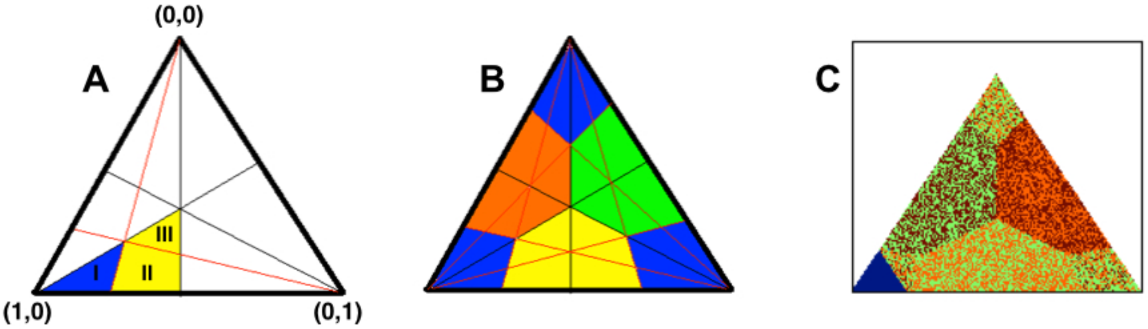

Figure 1. (A) Theoretical Greedy MAP policy plot for the region $\mathbf{p}_t^1 > \mathbf{p}_t^2 > \mathbf{p}_t^3$. (B) Theoretical Greedy MAP policy plot for the complete simplex. Blue: fixate location 1, 2 or 3. Yellow: fixate location 1 or 2. Green: fixate location 2 or 3. Orange: fixate location 3 or 1. (C) Simulated Greedy MAP policy. Blue: stop. Green: fixate location 1. Orange: fixate location 2. Brown: fixate location 3. When more that one actions is optimal in a region, the action is chosen randomly leading to the blotchy appearance.
